# Treatment of Cerebrospinal Meningitis

**Published:** 1905-11

**Authors:** 


### Treatment of Cerebrospinal Meningitis.

CHIEF STAFF SURGEON SHERWALD., (*Deutsche Med. Wochenschrift,* No. 35, 1905), reports a sporadic, typical and quite severe case in which all usual methods were employed, with only transient effects at the most. He then proposed daily inunctions of 2 drams unguentum Credé into the trunk, after cleansing with soap and alcohol. The effect was striking. With the very first inunction the clinical picture improved, and after four of them the patient was bright and cheerful and almost wholly free from subjective difficulties. As the treatment had not been altered in any other respect, there can be no doubt that the favorable turn in the disease is due to the silver salve. The author warmly recommends this simple and safe method to all practitioners.

Professor Bjorkman recommends the following treatment in cerebrospinal meningitis. Inunctions of unguentum Credé are given at first twice, and then once daily. At the same time the hair of the scalp is closely clipped, and moist borated gauze, drenched in a 1½ per cent. formaldehyde solution, acetanilid and 1-10 per cent. kresamine, is continually kept over the whole head from the root of the nose backward down to the neck and on the sides, leaving the ears free. The pack is covered with a cap of oil-cloth and renewed as soon as it becomes dry.—*Merck's Archives,* January, 1905.

Dr. Mitour contributes (*Bulletin gén. de. Therap. méd.,* July 15, 1905,) a paper to the silver therapy of cerebrospinal meningitis. For a woman with severe meningitic symptoms he ordered on the third day of the disease four inunctions of unguentum Credé. On the following day the convulsions ceased and the patient regained consciousness. Under two or three inunctions daily the temperature returned to normal on the ninth day of the sickness, and though the pulse was still a little rapid the general condition and appetite were excellent. Complete cure followed. The author also reports a case of febrile eclampsia cured by the same therapy.

